# Miniaturisation of Pressure-Sensitive Paint Measurement Systems Using Low-Cost, Miniaturised Machine Vision Cameras

**DOI:** 10.3390/s17081708

**Published:** 2017-07-25

**Authors:** Mark Kenneth Quinn, Emanuele Spinosa, David A. Roberts

**Affiliations:** 1 School of Mechanical, Aerospace and Civil Engineering, University of Manchester, Manchester M13 9PL, UK; em.spinosa@gmail.com; 2 Aircraft Research Association, Manton Lane, Bedford MK41 7PF, UK; droberts@ara.co.uk

**Keywords:** pressure-sensitive paint, PSP, machine vision, calibration, sensor development

## Abstract

Measurements of pressure-sensitive paint (PSP) have been performed using new or non-scientific imaging technology based on machine vision tools. Machine vision camera systems are typically used for automated inspection or process monitoring. Such devices offer the benefits of lower cost and reduced size compared with typically scientific-grade cameras; however, their optical qualities and suitability have yet to be determined. This research intends to show relevant imaging characteristics and also show the applicability of such imaging technology for PSP. Details of camera performance are benchmarked and compared to standard scientific imaging equipment and subsequent PSP tests are conducted using a static calibration chamber. The findings demonstrate that machine vision technology can be used for PSP measurements, opening up the possibility of performing measurements on-board small-scale model such as those used for wind tunnel testing or measurements in confined spaces with limited optical access.

## 1. Introduction

**Figure 1:**
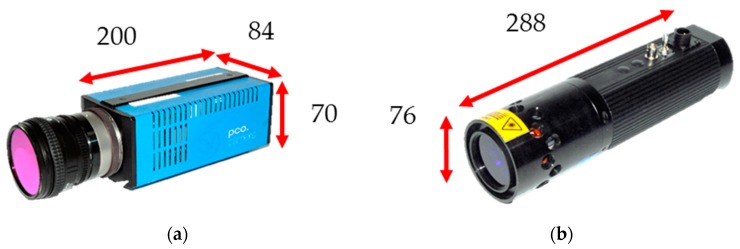
Typical PSP system components. All dimensions are in mm. (**a**) PCO.1600 camera; note that the dimensions do not include the lens; and (**b**) a high-power UV LED lamp from Innovative Scientific Solutions Incorporated.

Pressure-sensitive paint (PSP) measurements have been made to great effect in many areas of aerodynamic research. The ability to take field measurements of pressure has given researchers and aircraft designers new levels of insight into the flow physics they are investigating [1]. One area of interest is investigations of unsteady flows [2,3] where not only steady components of pressure but oscillating components and even aerodynamic modal shapes have been measured effectively. However, traditional (steady-state) PSP systems can often be large installations comprising of up to a dozen cameras with a similar number of lamps. Each of these camera and lamp pairs cost tens of thousands of pounds which, when added up to a total cost of a system, can easily run up to £500k. The typical size of traditionally used cameras (such as the PCO.1600 mod, manufactured by PCO AG, Kelheim, Germany) are 200 mm × 80 mm × 70 mm without lenses. This prohibits the use of such cameras when attempting to perform measurements inside wind tunnel models or in tight and confined spaces. Figure 1 shows typical PSP system components and demonstrates how the size of these components means that measurements in tight areas is often not possible. This problem is exacerbated when the specific requirement of the test is to have a particular view of an object in the flow.

The reason such cameras have been used is because of their exceptional recording/measurement characteristics. The PCO camera mentioned has active thermoelectric cooling in order to keep the sensor at a constant temperature, thereby maintaining a constant low noise level, which makes the camera very repeatable between tests. A typical property of all imaging systems is the link between increased temperature and increased noise level.

In recent years there has been a significant increase in the capability of miniaturised cameras. This is evident from the consumer market of smartphone cameras, with the average resolution of a smartphone sensor increasing threefold from 2.4 megapixels in 2007 to over 9 megapixels in 2015 [4]. The recent rapid advancement of imaging technology has made now a prudent time to investigate the possibility of using smaller, significantly cheaper, cameras to perform PSP measurements. Many of the cameras investigated within this study are machine vision cameras, such as the Teledyne Dalsa Genie Nano; however, despite their diminutive size they often possess camera qualities, such as quantum efficiency, which rival or even exceed traditional scientific measurement devices. With this in mind, this study aims to investigate the imaging characteristics of machine vision cameras, compare their characteristics to scientific, cooled cameras and demonstrate their suitability for PSP measurements by performing static calibrations.

## 2. Background

### 2.1. PSP Technique

The PSP technique has been used from low-speed flows [5] to ultra-high-speed transient flows [6] and everything in between. There have been many excellent descriptions and introductions to PSP, so only a brief overview will be given here (readers are encouraged to investigate the review paper by Gregory et al. [7] and the comprehensive book by Liu and Sullivan [8]).

#### Basics

**Figure 2:**
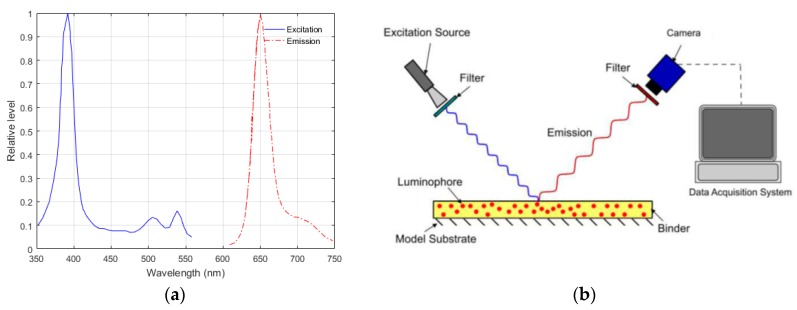
(**a**) Absorption spectra and emission spectra of a PSP containing PtTFPP and (**b**) basic set-up of a PSP system [9].

PSP is a technique based on the oxygen quenching of luminescence that allows field measurements of pressure on a surface. The active molecule of PSP is known as a luminophore. The luminophore is dissolved in an oxygen-permeable binder material with solvents and other additives and sprayed onto a suitably prepared substrate. PSP absorbs light of a specific wavelength (usually around 390 nm) and is excited to a higher electronic state. The excited luminophore molecule then gives this energy away either by emitting a longer wavelength of light (usually around >600 nm) or via contact with a deactivating/quenching molecule (often oxygen). The molecule can also be deactivated by radiationless methods. The probability of radiationless deactivation is increased with temperature, a property known as thermal quenching. By measuring the amount of light emitted, one can measure the amount of oxygen present and, in turn, the pressure. A typical spectral plot for PSP is given in Figure 2a. The typical measurement system for PSP incorporates a narrowband excitation source or a broadband excitation source with appropriate filters and an imaging device with appropriate filters (Figure 2b). The filters are used to separate out the two signals.

The basic PSP system is known as a radiometric PSP system. This system measures the intensity of light outputted by the paint and relates it to a reference condition. The relationship between this reference condition (sometimes called a wind-off condition) and the test condition is expressed using a modified version of the Stern-Volmer equation given below:

(1) $$\frac{I_{ref}}{I}=A\left(T\right)+B\left(T\right)\frac{P}{P_{ref}},$$

where I is the pixel intensity, P is the pressure, A and B are coefficients which are functions of temperature (T) and the subscript ref denotes a reference condition. This equation is applied pixel-wise to the PSP data and, given a suitable calibration, allows for calculation of the pressure at every pixel. Unfortunately, PSP also exhibits temperature sensitivity (through the coefficients A and B) which must be quantified in order to understand the limitations of the technique.

### 2.2. Imaging Characteristics

The EMVA 1288 standard [10] describes and details the many different metrics for image and camera quality that can be used when deciding on which camera technology to use and how to benchmark performance of imaging systems. In this work, only parameters relevant to PSP measurements (based on a representative acquisition scheme) were investigated. The following metrics were measured for each machine vision camera and compared against a PSP industry-standard camera—a PCO.1600:

- Dark signal (Dark noise)
  ○ Specifically dark signal vs. exposure time
- Temporal stability
  ○ Measured over a large number of images
- Linearity
  ○ Measured over a range of exposure times for a constant input light source
- Signal-to-noise (SNR) ratio
  ○ Measured at different levels of exposure and against number of images averaged

#### 2.2.1. Dark Signal

Dark signal is the signal present in the image delivered by a camera in the absence of light. This is known to be temperature-dependent and, depending on the application, can be very significant for machine vision cameras which do not have any active cooling present. The values recorded as dark signal should be subtracted from the measurement value before any subsequent processing takes place.

#### 2.2.2. Temporal Stability

As machine vision cameras do not have active cooling, they may heat up during subsequent exposures and change the values present in the image. This heating can be caused by the operating environment or due to self-heating from internal electric currents. As the sensor or camera body heats up, there may be spatial changes across the sensor which could be significant for PSP imaging and therefore needs to be quantified.

#### 2.2.3. Linearity

The response of a camera to incoming photons should, ideally, be linear in that twice the number of photons per given area should result in twice the signal output. This is usually true over the majority of the dynamic range of a camera; however, at the edges this assumption tends to break down and the response becomes non-linear. The output from the camera at different exposure times can be plotted and, using a least-squares regression, have a linear curve fitted to it. The maximum deviations from this curve are used to estimate the non-linearity of the camera response as a single parameter:

(2) $$NL=\frac{\left|\delta I_{+ve}\right|+\left|\delta I_{-ve}\right|}{I_{max}}\times 100,$$

where NL is the non-linearity in percent, $\delta I_{+ve}$ is the maximum positive deviation from the least-squares curve, $\delta I_{-ve}$ is the maximum negative deviation from the least-squares curve and $I_{max}$ is the maximum signal (12 bit = 4096). It is important for sensors used for quantitative imaging have a good linearity otherwise a more complicated lookup table approach must be used.

#### 2.2.4. Signal-to-Noise Ratio

This is an extremely important parameter for imaging as it demonstrates how much of a reading can be attributed to noise and how much can be apportioned to the measured quantity. SNR is often quoted in decibels:

(3) $$SNR=20\times \log_{10}\frac{\mu -\mu_{dark}}{\sigma },$$

where μ is the pixel exposure, $\mu_{dark}$ is the dark level for this given exposure and σ is the standard deviation of the pixel exposure levels. The SNR can be improved by averaging multiple images, something which is often done in PSP tests and is investigated in this study.

## 3. Imaging Devices

Traditional (charge-coupled device) CCD cameras have a pixel well that collects electrons and then the charge of those electrons is then converted to a voltage by means of a charge amplifier after they have been shifted out of the pixel well. These voltages are then digitised using an analog-to-digital converter (ADC).

By contrast, complementary metal-oxide-semiconductor (CMOS) cameras have the charge conversion built into each pixel individually and so they output a voltage rather than a charge. This has the advantage of being significantly faster (as the conversion happens in parallel); however, tolerance limitations on the electronics inbuilt into each pixel means that CMOS cameras are more likely to suffer from fixed-pattern noise and non-uniform sensor response. Despite this traditional limitation, tolerances on modern electronics have improved such that this difference is undetectable in all but the most sensitive experiments. As such, all of the machine vision cameras tested within this study are of CMOS architecture, whereas the PCO.1600 is based on CCD architecture.

The general specifications of the cameras investigated in this study are given in Table 1. The PCO.1600 camera was used as a benchmark for the machine vision cameras as this is a standard camera used for both academic and industrial PSP measurements. During set-up the PCO camera was set to use the 10 MHz ADC clock with only one ADC as this has been found to give the best quality output. One of the main things to notice when comparing the cameras is that the machine vision cameras are significantly smaller (with the Ximea XiMU camera being an order of magnitude smaller); however, they do have a lower bit depth compared to the PCO. The Ximea camera also only has a progressive scan shutter, meaning it is subject to scan lines being present in the image and is susceptible to rolling shutter effects; however, as these cameras are only to be used for steady state PSP measurements in this study, this does not pose a significant issue. In measurements where aeroelastic phenomena may be present, the use of a progressive-scan cameras may not be suitable.

### Software

Software to control the machine vision cameras was written in LabVIEW using the Vision Acquisition Toolbox, taking advantage of the GenICam standard where possible. This standard allows for the quick development of camera-control software that is easily portable from one camera to another [11]. Unfortunately, as the Ximea camera is USB 2.0, it is not able to communicate using the GenICam standard; however, Ximea have developed a LabVIEW SDK which allows for the creation of similar software to that used by the GenICam controlled cameras. All of the possible parameters that could be controlled on board the camera required investigation to ensure that no on-board processing of the images was enabled by default. This was achieved by using National Instruments Measurement & Automation Explorer (NIMAX) and then programmatically setting these parameters using property nodes within LabVIEW. The following parameters were required to be set for the machine vision cameras (note that the names of the parameters are exactly as they are called in LabVIEW):

- BlackLevel = 0
  ○ Sets the threshold, below which, the camera reports a zero level. This was set equal to zero, thereby maximising the measurement range available.
- PixelFormat = 12 bit
  ○ Default bit depth is 8 bit for all cameras but was set to 12 bit for greater resolution.
- ExposureAuto = off
  ○ Otherwise the camera will adjust the exposure time to “correct” the exposure.
- GainAuto = off
  ○ Otherwise the camera will try to automatically adjust the gain to “correct” the exposure.
- Gain = 1
  ○ So that this value is set and controlled.
- AcquisitionFrameRate = as required
- ExposureTime = as required

Once all of these parameters were set and the camera reported that it was ready, data was captured at the specified frame rates and exposure times in a producer-consumer architecture and saved on a solid-state hard drive inside the controlling PC. The PCO camera was controlled using Camware, the standard PCO imaging software, with all data being saved directly to disc.

## 4. Methodology

This section will explain the methodology used to benchmark the cameras and perform PSP calibrations.

### 4.1. Camera Benchmarking

When measuring the response of the cameras, they were left powered up for approximately 5 min to reach a stable operating temperature before acquiring data. There appeared to be no measurable change between leaving the cameras powered up for 5 min or several hours. The Genie Nano and Ximea XiMU cameras can both report their temperature to the controlling PC and no differences were found in their temperature after being powered up for longer than 5 min.

Dark signal measurements were made with the camera in total darkness with different exposure times (0.1, 1, 10, 50, 100, 200, 500 and 800 ms). For each dark value estimation experiment, 30 images were taken so that statistical quantities could be estimated. The average values of exposure and the standard deviation across the images were taken (and averaged to produce a single numerical output) and plotted against exposure time.

Temporal (and thereby thermal) stability was measured by taking 1000 dark images (in the same manner as mentioned above) with 10 ms exposure. These results allow for the visualisation of the stability of the cameras and to see if they are significant when compared to the range of the sensor.

Linearity measurements were taken by using a uniform white light source made up of the backlight for a laptop; all other sources of ambient light were switched off or removed. The light source was left energised for several minutes to reach thermal equilibrium. The cameras had no objective lenses fitted and were placed at a distance away from the screen such that they had very similar exposure times (see Table 2) for an output of approximately 95% of their dynamic range. The exposure time was then reduced by 0.1 ms until a minimum exposure level was reached or an exposure time of 200 ns. 30 images were taken at each condition and averaged before processing.

**Figure 3:**
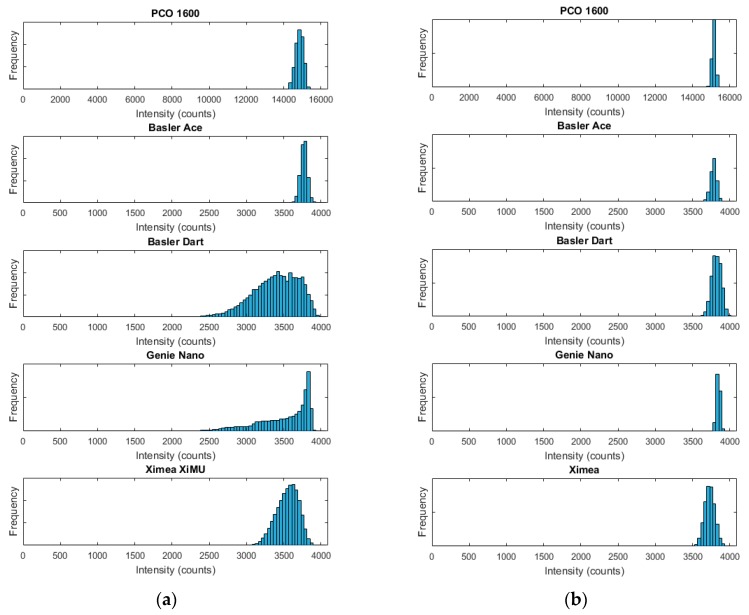
(**a**) Histogram output of complete image and (**b**) histogram output of 300 × 300 pixel region of interest in centre.

Finally, the SNR was measured using the same light source as above. The exposure levels were then matched between the cameras by matching the maximum values in the histogram (below the saturation level), as shown in Figure 3. This set-up is representative of the use of such cameras for PSP measurements. The exposure times used were the same as the linearity maximum exposure times given in Table 2.

**Figure 4:**
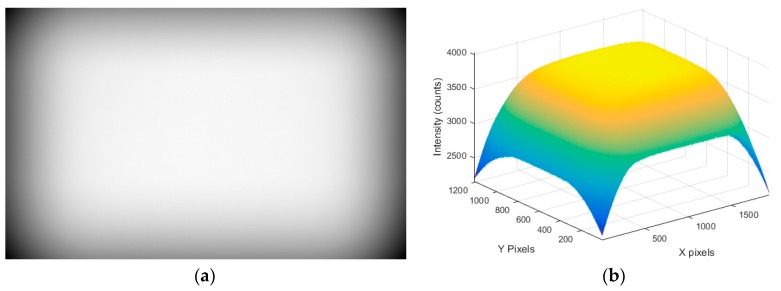
(**a**) Raw image from Genie Nano at 100% brightness and (**b**) surface visualisation of vignetting.

The dark signal levels measured at the maximum exposures shown in Table 2 were subtracted from the SNR images before SNR estimation took place. As the levels of vignetting differed, matching the histogram patterns was not possible, as can be seen in Figure 3a. In order to solve this issue, a region of interest (ROI = 300 × 300 pixels) centred on the maximum brightness value was extracted for further processing. As can be seen in Figure 3b, this approach matches the histograms much more evenly. A typical example of this from the Genie Nano camera without an objective lens is shown in Figure 4.

**Figure 5:**
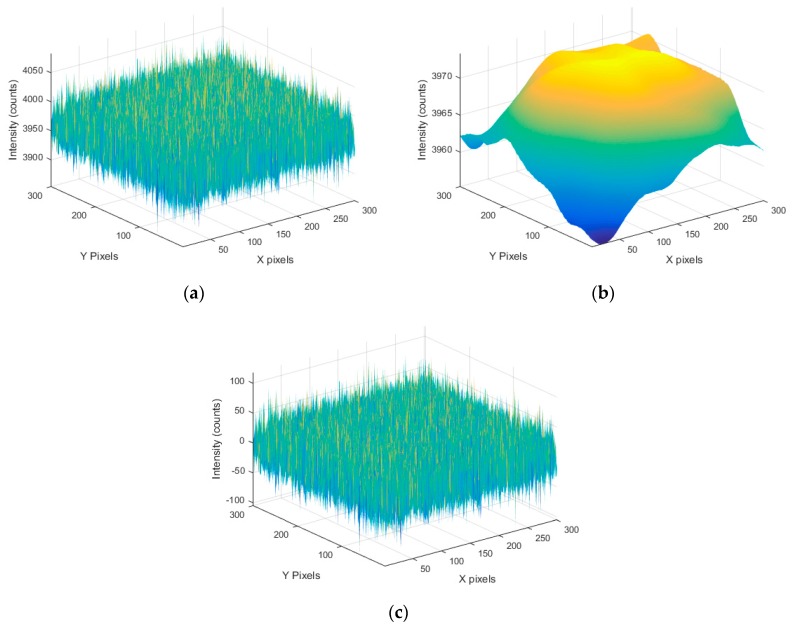
(**a**) Averaged ROI from Genie Nano at 100% brightness; (**b**) 20-pixel radius Gaussian-filtered averaged ROI; and (**c**) averaged ROI with Gaussian-filtered profile subtracted.

In order to get a more representative estimate of the SNR, all of the captured images at each brightness level were averaged and the ROI was extracted (Figure 5a). This averaged ROI was then Gaussian-filtered to remove the noise in the shape (Figure 5b). Finally, the filtered shape was then subtracted from the raw data giving a zero-mean value for the noise without any fixed pattern (Figure 5c).

### 4.2. PSP Calibration

Static calibration of the PSP sample took place in an in-house-designed calibration chamber detailed in Section 5.3 with other relevant hardware described in Section 5. During the PSP calibration, the effects of three temperatures on the PSP sample were investigated: 273, 293 and 313 K, which were held constant (to within T±0.5 K) while the absolute static pressure in the chamber was varied from P=10−150 kPa in 10 kPa steps. At each condition 30 images were taken so that they could be averaged to increase the signal-to-noise ratio. The reason for choosing 30 images is shown in Section 6.4.

## 5. Experimental Set-Up

The PCO.1600, Basler Ace and Genie Nano cameras were mounted on tripods while the Basler Dart and Ximea XiMU were clamped in a post-mounted clamp (as they do not have tripod mounts) at a similar distance from the target.

For PSP calibration testing the PCO camera had a 24 mm F2.4 Nikon F-mount lens, while the Basler Ace and Genie Nano cameras both used a 50 mm F2.0 C-mount lens. The Ximea XiMU and Basler Dart cameras used a 1.8 mm F1.8 S-mount lens with a locking ring to secure focus. All of the tests were performed with the aperture as wide open as possible and the images were set to be at approximately the same real-world scale. For all other testing (camera benchmarking) none of the cameras had lenses fitted.

In order to compare camera SNR and dark signal performance all of the images were captured at 5 Hz or as fast as could be captured given the exposure time. The thermal stability of each camera was demonstrated by capturing 1000 images as fast as the camera could, thereby driving it as hard as possible. However, during sample PSP calibrations, images were taken at 20 Hz or as fast as the camera was able to capture. This was chosen as it is more likely to represent a real-world test of the cameras rather than a purely academic one.

### 5.1. Filters

As PSP measurements require spectral separation of the excitation and emission signals, filters were required on the lenses of the cameras. The filters used in this experiment were Schott RG645 long-pass filters (manufactured by Galvoptics, Basildon, United Kingdom) which were mounted on the front of the lenses of the cameras. This filter is ideal for PSP experiments as it has an optical density (OD) of OD≥5 up to 600 nm and above 95% transmission thereafter.

### 5.2. Lights

Illumination for the linearity and SNR tests came from a modified laptop display powered by a stable, digital bench power supply. The display had the LCD removed and the internal circuitry was stripped back to light controller. On-board the laptop the display brightness was originally controlled via pulse width modulation (PWM); however, the PWM circuitry was replaced with a current-limiting resistor and a stable, digital DC power supply which could be programmed to set voltages and currents.

**Figure 6:**
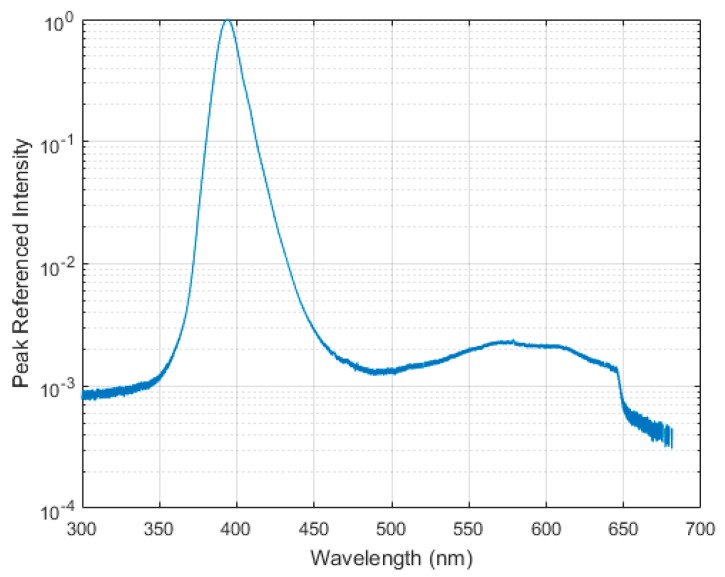
UV LED lamp spectrum.

Illumination for the PSP measurements came from the same in-house-built UV LED array used by Quinn and Kontis [9] which was made up of 144 Bivar 395 nm UV LEDs. As this light does not contain any temperature control or forced cooling, it was left to settle to a running temperature at a fixed voltage before PSP data acquisition began. During this time the PSP sample was not exposed to the light to avoid excessive photodegradation. The spectrum of the LED lights was measured using a Thorlabs CCS200/M Spectrometer to ascertain if there was any significant output in the red part of the spectrum, a common issue with high-power UV LEDs. Figure 6 shows that there is very little output in the red region of the spectrum (approximately 500 times lower in magnitude than the 395 nm UV peak), making these lights suitable for PSP measurements.

### 5.3. PSP Calibration Chamber

**Figure 7:**
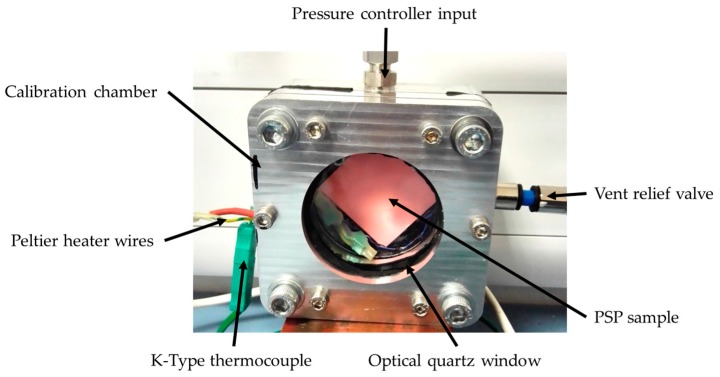
PSP calibration chamber.

This system consists of an in-house-designed pressure chamber with a quartz glass window and a thermoelectric Peltier element, as shown in Figure 7.

The pressure was controlled using a GE Druck Pace5000 pressure controller which, given a negative and positive pressure input, can control pressure with an accuracy of ±22 Pa over a range of 0–3.5 bar absolute. The repeatability of the pressure controller is to within the same accuracy (±22 Pa). The Peltier device was connected to a lab power supply to either heat or cool the PSP sample depending on the direction of current flow. Temperature was measured using a K-type thermocouple and an AD594 amplifier connected to a differential measurement channel of a National Instruments myDAQ. Setting the temperature at the beginning of the PSP pressure sweep was accurate to within 0.25 K.

### 5.4. PSP Sample

An aluminium plate was cleaned with acetone and then sprayed with a matte white paint before being over-sprayed with ISSI UniFIB PSP [12] using a modeller’s airbrush. UniFIB is a proprietary PSP sold by ISSI which incorporates PtTFPP as the photoactive molecule. The spectra for this luminophore is given in Figure 2a. The sample was then baked in an oven to drive off moisture and evaporate the solvents used in the paint. The PSP sample was placed in the calibration chamber and held in contact with the Peltier device with heatsink paste to ensure good thermal conductivity.

## 6. Camera Benchmark Results

This section will present and discuss the results of the camera benchmarking tests.

### 6.1. Dark Signal

**Figure 8:**
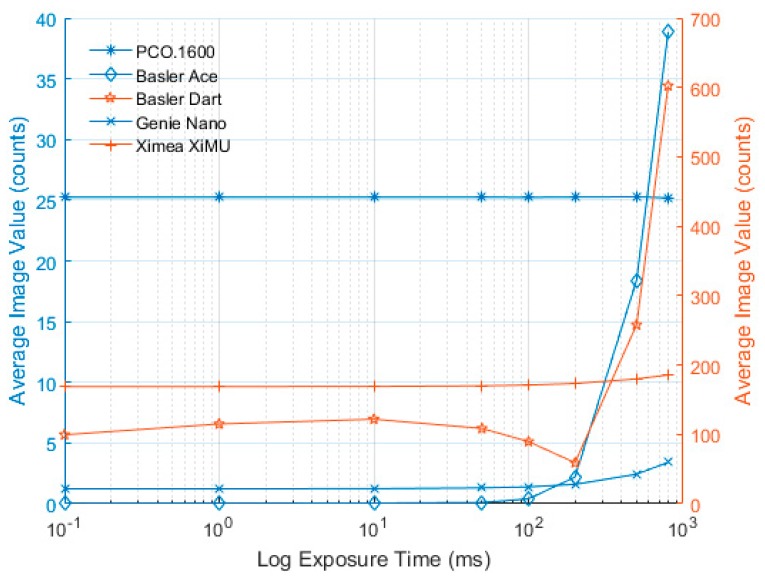
Mean dark exposure value vs. log exposure time for all cameras. PCO.1600, Basler Ace and Genie Nano are plotted against the left y axis and Basler Dart and Ximea XiMU are plotted against the right y axis.

**Figure 9:**
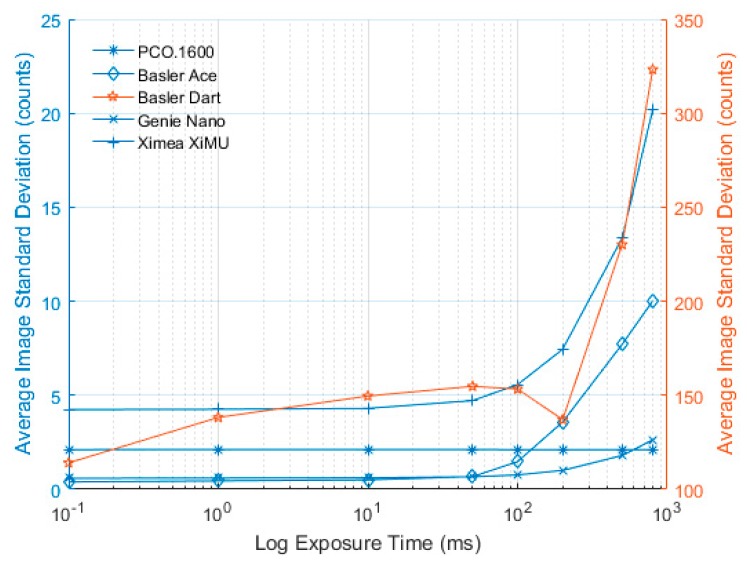
Image standard deviation vs. log exposure time for all cameras. PCO.1600, Basler Ace, Genie Nano and Ximea XiMU are plotted against the left y axis and Basler Dart is plotted against the right y axis.

The dark signal measured for each camera at different exposure times is presented in this section. In order to be able to compare the values from 12-bit and 14-bit cameras on the same figure, the values recorded by the PCO.1600 were scaled to a 12-bit scale (i.e., divided by four). Also as the values vary significantly between cameras, plots were created where different cameras were plotted on different vertical axes of the same figure according to the colour in the legend. The PCO.1600 camera shows excellent stability for different exposure times, with no differences distinguishable between images either in terms of mean value (Figure 8) or variability (Figure 9).

In Figure 8, the Basler Ace shows extremely good dark signal characteristics and very low noise levels until 100 ms exposure time, after this time the dark value and standard deviation seem to increase significantly. A recommendation for this camera would be to keep the exposure time below 100 ms wherever possible.

The Basler Dart exhibits an unusual dark signal profile both in terms of absolute level (Figure 8) and variability (Figure 9). This measurement was repeated multiple times to ensure that the results were reliable and the same pattern was demonstrated. Due to the way the camera reads out its data (the actual exposure is at the end of the frame but the sensor is active throughout), the dark signal is actually more susceptible to frame rate than exposure time. No other camera exhibited this behaviour when acquisition rates were changed and the tests were repeated. A recommendation for this camera would be that the exposure time is kept below 100 ms wherever possible and the data should be captured as fast as possible (maximum frame rate is 60 fps). It should also be noted that the mean values and standard deviation are significantly higher than those for the Basler Ace.

The dark signal characteristics of the Genie Nano show that the dark signal is very low when the exposure time is below 10 ms and only increases slightly when using longer exposures. The values presented here are comparable to the Basler Ace; however, when using longer exposures the Genie Nano performs significantly better.

In Figure 8, the Ximea XiMU camera shows very similar trends to the other cameras with the notable exception that the initial mean value is significantly higher than for the other cameras. However, the standard deviation is relatively low (Figure 9) and the uniformity is very good; therefore, the mean value can be subtracted when performing PSP tests, with no significant impact on the results apart from reducing the useable range of the 12 bit ADC value.

As expected, all of the cameras (without cooling) exhibited an increase in standard deviation across the image, clearly shown in Figure 9. However, the level of increase for the majority of the cameras (Dart and possibly XiMU excluded) is relatively low. In addition, there is almost no increase detected for exposure times below 50 ms, indicating that if exposure times can be made short enough (with sufficient light power) these cameras may be suitable for PSP measurements. It is also worth noting that the PCO.1600 camera, with its closed-loop temperature control, exhibits no measurable change in either mean dark value or standard deviation with exposure time.

**Figure 10:**
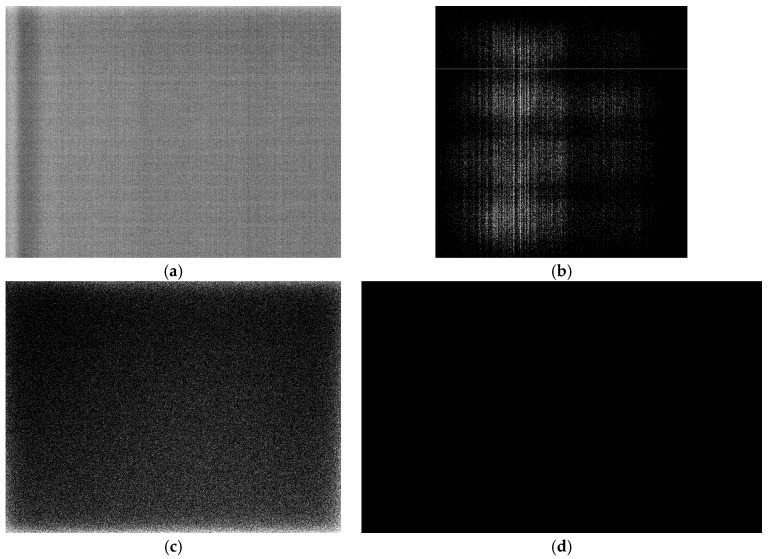

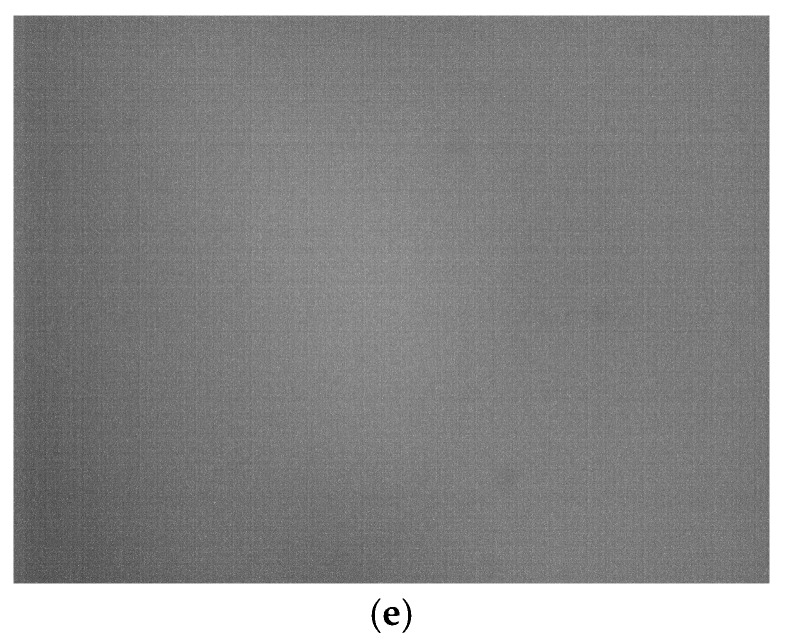
Fixed-pattern noise found in averaged 100 ms dark exposure images. (**a**) PCO.1600 image scaled between 92–104 counts, (**b**) Basler Ace image scaled between 0–2 counts, (**c**) Basler Dart image scaled between 0–24 counts, (**d**) Genie Nano image scaled between 0–1 counts and (**e**) XiMU image scaled between 163–178 counts.

Very little significant fixed pattern noise can be found in any of these images as seen in Figure 10. Note that in this figure all of the images are presented on a different scale so as to make them visible. The PCO.1600 shows a strange band at the left hand side of the image (Figure 10a); however, this is small in magnitude and can be subtracted via the dark image. The Basler Ace has very good uniformity such that the values shown are only between 0 and 2 counts (Figure 10b). The Basler Dart camera shows that one side of the image (the lower edge) has much higher non-uniformity than the other regions (Figure 10c). This is likely due to the location of the electronics in relation to the sensor, making this part of the chip much hotter. The Genie Nano shows remarkable uniformity (Figure 10d) in that there is no evidence of fixed-pattern noise at all. Finally, the Ximea XiMU (Figure 10e) shows some fixed pattern noise at a relatively high exposure level (as would be expected from Figure 8). However, the uniformity of this is rather good and there is little evidence of significant scanlines present in the final image, something which is potentially a problem for progressive-readout cameras.

### 6.2. Long-Time Response

The long-time response of the cameras showed some surprisingly good results, especially given that the machine vision cameras are not cooled. All of the cameras, with the exception of the Basler Dart, showed no deviation across the 1000 images greater than 1.5 counts, with the standard deviation of pixels within the images also remaining constant. If the values for the PCO.1600 camera are divided by four (to bring them into a comparable range for the 12-bit machine vision cameras) the values are very much in line in terms of standard deviation, implying that by this metric there is little to differentiate between the cameras.

**Figure 11:**
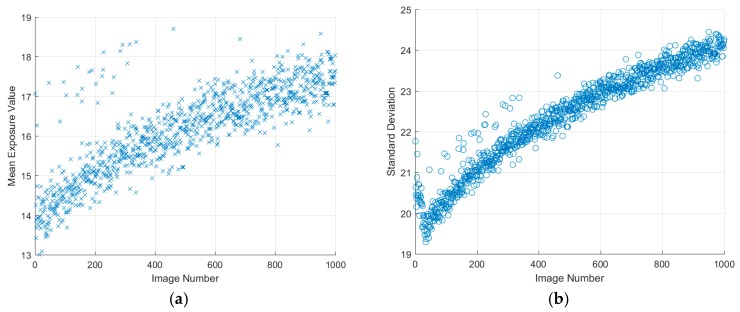
Basler Dart long-time response over 1000 images (**a**) mean image value and (**b**) image standard deviation.

The Basler Dart did show some dependency on the number of images taken, as can be seen in Figure 11. However, given that only a relatively small number of images are taken during a PSP data point, this change may not be significant. On the other hand, it does imply that the thermal stability of this camera could be called into question for PSP measurements.

### 6.3. Linearity

**Figure 12:**
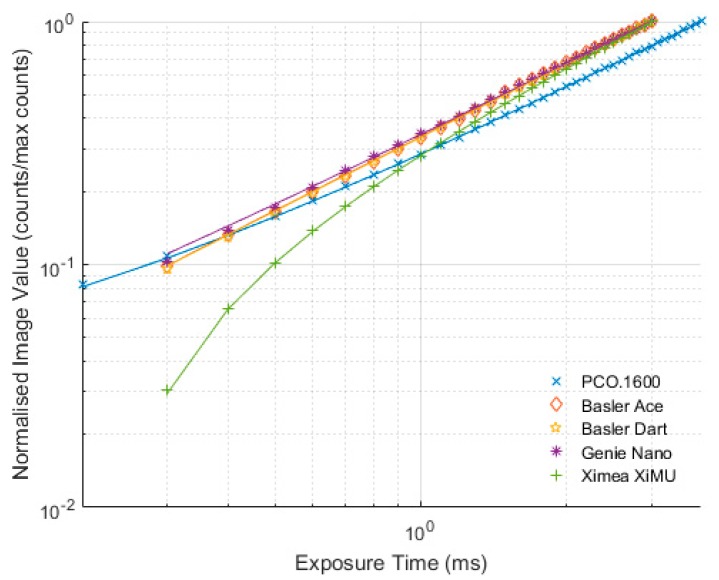
Normalised exposure level vs. exposure time for different cameras.

The linearity of the cameras was measured by changing exposure time for a fixed light source. In Figure 12 all of the cameras are shown on the same plot with the vertical axis value being normalised by the maximum signal measured; allowing all cameras to be visualised at once. The solid lines between the data points represent a linear least-squares curve fit. The numerical values of the curve fit parameters (gradient and offset), the coefficient of determination (R^2^) and the percentage non-linearity, as per Equation (2), are shown in Table 3.

All of the cameras showed linearity with very predictable trends. The most illuminating result is the non-linearity percentage which suggests that the XiMU camera actually has better linearity than the PCO.1600. The PCO.1600 camera has a published non-linearity of better than 1%, something the camera easily exceeded here [13]. The published value of non-linearity for the XiMU is better than 0.2% [14], something which is almost matched in these tests. None of the other manufacturers publish data on the non-uniformity of their cameras.

### 6.4. Signal-to-Noise Ratio

**Figure 13:**
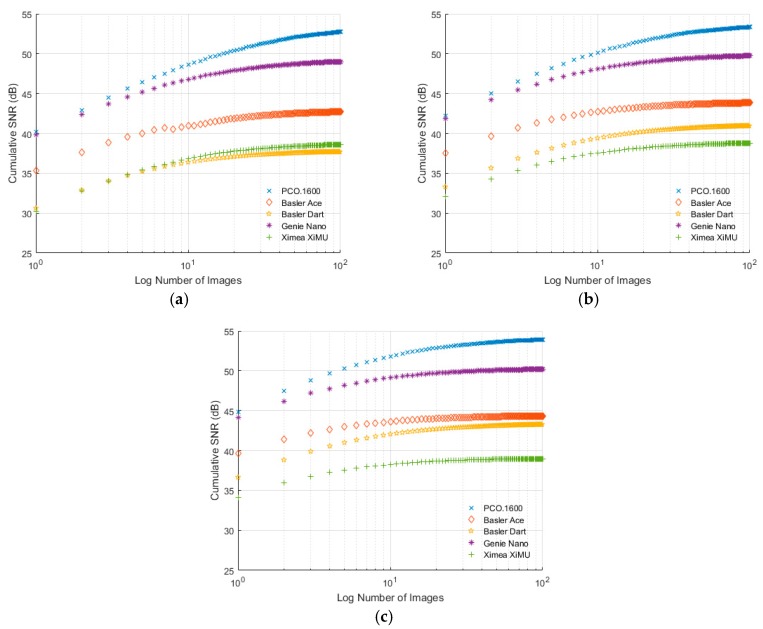
SNR for the different cameras measured with (**a**) 25% brightness, (**b**) 50% brightness and (**c**) 100% brightness.

Figure 13 shows the SNR results for all of the cameras at different exposure levels vs. the number of images averaged. From this figure it is clear that the PCO.1600 performs best out of the cameras tested. PCO do not publish SNR data for their cameras; however, from Figure 13 it appears that, when a suitable number of images are averaged, this value can be as high as 54 dB. The second best performer is the Genie Nano, which has a published SNR of 43.9 dB [15]. This is close to the value recorded here from a single image, but if multiple images are averaged this value can reach 50.2 dB. The performance of the Basler Ace and Basler Dart at 100% brightness (Figure 13c) is very similar; however, Figure 13a,b show that the Basler Dart has a very strong dependency on the level of incoming signal, something not ideal for PSP measurements. The Basler Ace has a published SNR of 40.8 dB [16], which is slightly above the single-image value measured here of 38.5 dB; however, if multiple images are averaged, this value reaches 44.4 dB. Finally, the Ximea XiMU camera has a published maximum SNR of 38 dB [14] which is above the single-image measured values but is reached by averaging only six images. This value increases to 39 dB if more images are averaged.

It is interesting to note from Figure 13 that, apart from the Basler Dart, the cameras tested exhibit a relatively low SNR dependency on incoming signal strength, with all cameras asymptotically reaching the same value of SNR, regardless of exposure level. This is not that surprising given the $1/\sqrt{N}$ nature of random noise when multiple samples are averaged, but it is useful to know to create a best practice guide for performing PSP tests with these cameras where wind tunnel time is expensive.

### 6.5. Camera Benchmark Conclusions

The following conclusions can be drawn from the camera benchmark tests:

- The PCO.1600 camera performs significantly better than the machine vision cameras in all areas of the measurement, as would be expected for the significantly more expensive scientific-grade camera.
- The long-time response of all of the cameras appears to be acceptable for scientific measurements.
- The linearity of all the cameras is suitable for quantitative imaging. The XiMU camera performance is very impressive in this respect.
- The SNR measured from the cameras is in line with the manufacturers’ datasheet but can be improved by averaging multiple images.
- Only the Basler Dart shows a dependency of noise and image quality on frame rate.

The following guidelines were developed from this benchmark test and subsequently followed for the PSP calibration testing:

- The exposure time of the machine vision cameras should be kept to 10ms or below to avoid significant increase in dark current and noise.
- 30 images should be averaged to improve the SNR of each data point (further increasing this value does not have a significant impact).
- The Basler Dart camera should be run as fast as possible.

## 7. PSP Calibration Results

**Figure 14:**
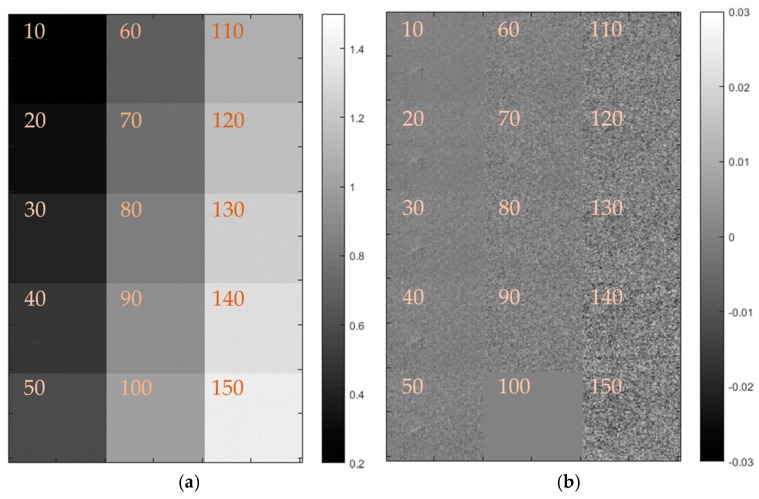
Monochrome calibration montage from Ximea XiMU camera at 293 K. The values marked in the figures relate to the pressure in kPa (**a**) Intensity ratio with increasing pressure and (**b**) deviation from mean. Each square is approximately 25 mm × 25 mm.

Each set of 30 images at each condition were averaged and the ratio was then taken with the reference condition (100 kPa at 293 K) for that camera. A montage of typical divided images is shown in Figure 14 below (note that only the PSP sample is shown, not the whole image) to demonstrate the uniformity of both the sensor and PSP response.

**Figure 15:**
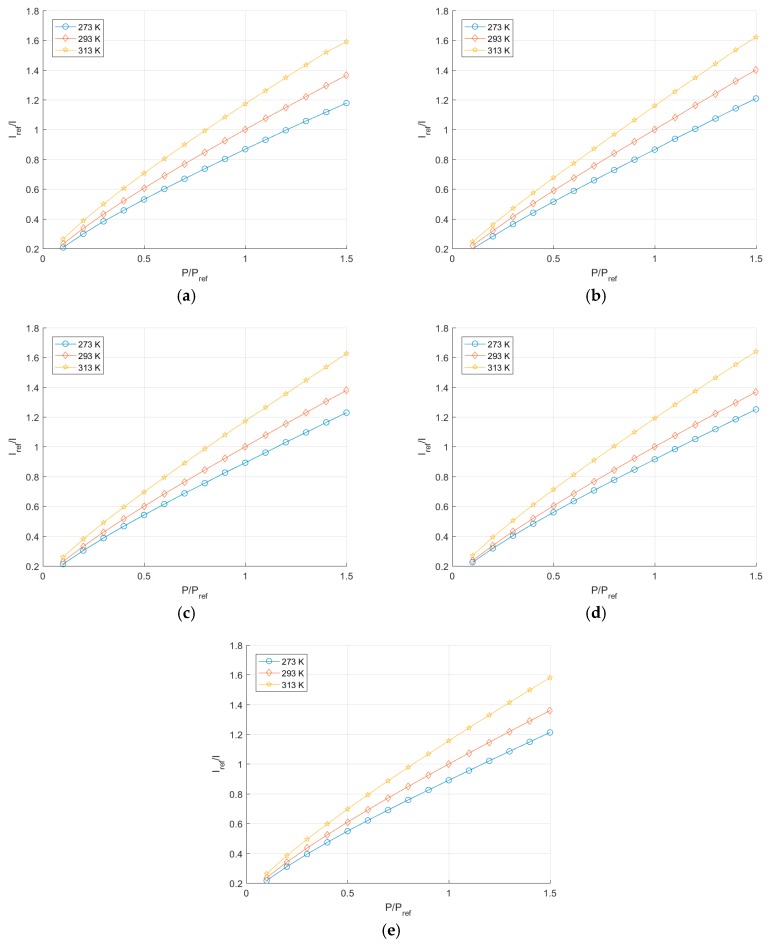
Stern-Volmer plot for (**a**) PCO.1600, (**b**) Basler Ace, (**c**) Basler Dart, (**d**) Genie Nano and (**e**) Ximea XiMU.

The deviation from the area average is shown also, demonstrating that there is no repeating pattern present. The signal-to-noise ratio is clearly decreased as the pressure increases, something to be expected given the nature of oxygen quenching reducing the luminescent output of the PSP. It is worth stating that the cameras all responded in the expected manner, with the quenching due to oxygen clearly visible in the results, demonstrating the usefulness of such imaging sensors for these measurements. Plots are shown for all of the cameras in Figure 15, but are not discussed in detail as they all respond in the expected fashion and give similar results.

### 7.1. Temperature Sensitivity

**Figure 16:**
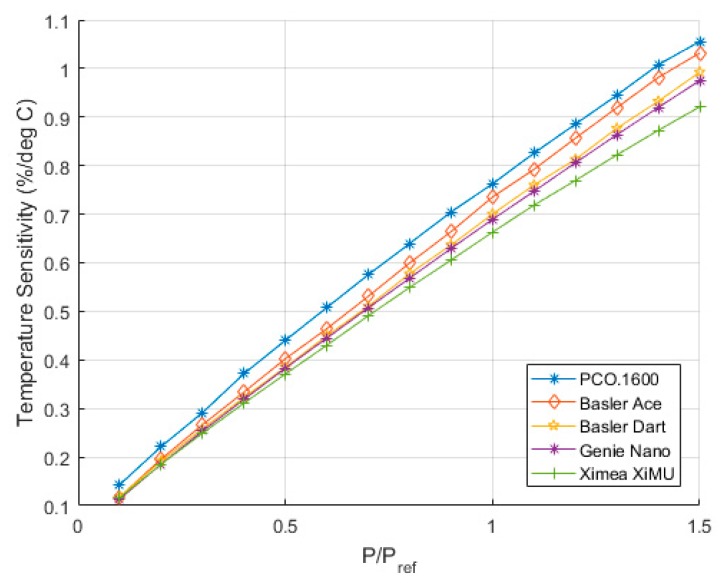
Temperature sensitivity of the UniFIB paint as measured using the different cameras tested.

The temperature sensitivity measured by the cameras was quantified by taking the average difference between the intensity responses for different temperatures as a function of pressure. The comparison between the different cameras is shown in Figure 16. The results in this figure are consistent with the datasheet for this paint [12]. The quoted temperature sensitivity for the ISSI UniFIB paint is $T_{sens}=0.4$%/C @ 100 kPa. It is worth mentioning that the actual temperature sensitivity measured here is larger than the quoted values from the datasheet, but this is susceptible to numerous factors such as spraying technique of the paint, exact filters used, the excitation wavelength and even the humidity on the day. As all of the cameras give results which are consistent with each other, it is reasonable to believe that this is the temperature sensitivity of this particular sample. The following section discusses the calibration characteristics of the PSP sample obtained using all of the test devices.

### 7.2. Comparison of Cameras

The calibration parameters calculated for each camera and temperature combination are shown in Table 4. All of the parameters calculated fall within the range of values given in [8] for paints with this luminophore More specifically, the UniFIB paint is quoted as having a pressure sensitivity of 𝑃𝑠𝑒𝑛𝑠 = 0.8%/kPa @ 293 K. This value agrees well with the B calibration parameters which are all centered around 0.8%/kPa @ 293 K.

**Figure 17:**
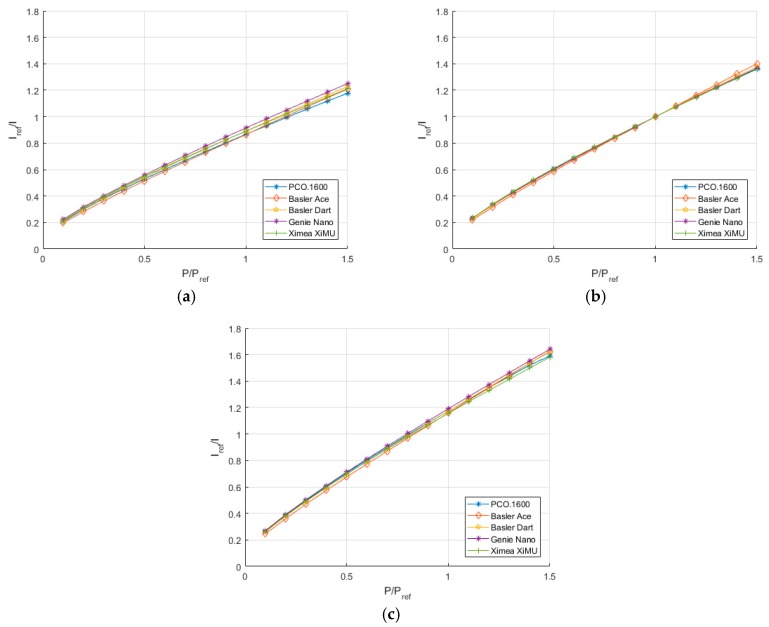
Comparison of calibration plots between cameras at (**a**) 273K, (**b**) 293K and (**c**) 313K.

Figure 17 shows the comparison between different cameras at constant temperature and allows one to compare the relative performance when the PSP should behave identically. In these figures there appears to be no systematic trend between the cameras. It is reasonable to expect that the difference between the different calibration curves is down to the uncertainty when maintaining a constant temperature. It is unlikely that, given the results of the linearity test, the difference in calibration is due to non-linearity effects.

### 7.3. Uncertainty Analysis

In order to estimate the uncertainty in the PSP measurements, it was decided to quantify the uncertainty in the calibration parameters as presented (A and B). This was performed by rearranging the Stern-Volmer equation as a function of A and B respectively and evaluating the sensitivity coefficients with respect to every parameter in the equation [17]. The estimation of temperature-induced error (i.e., by not holding the temperature perfectly constant) was evaluated by using the results from Figure 16 as a reference for the sensitivity to temperature. The results are evaluated at the standard reference condition (P = 100 kPa, T = 293 K). In order to quantify the image ratio uncertainty, the reference image was divided by the image at P = 10 kPa and T = 293 K conditions. The standard deviation of the image ratio (I100kPaI10kPa) was scaled to give 95% confidence (i.e., multiplied by 1.96). The results were then added together using the root-sum square method to give an overall uncertainty in the coefficients as shown in Table 5.

The dominant error in this experiment is the temperature control of the Peltier device used on the PSP sample and, as the B coefficient shows more dependency on temperature, it exhibits a larger uncertainty. The image ratio uncertainty is one order smaller than the temperature sensitivity for all cameras, but does introduce an appreciable difference in the calibration coefficients. Despite the temperature-induced uncertainty, these results show that all of the measurements taken by these cameras show low enough variability to be used for credible PSP measurements.

## 8. Conclusions

PSP measurements have been taken using small, affordable machine vision cameras. The results show that machine vision technology has advanced to such a state that it can now be used for quantitative measurements demonstrating that a PSP system can be implemented for under £1000. Cooled, high bit depth cameras such as the PCO.1600 have superior imaging performance but come with the caveat of larger cost and size. Of the machine vision cameras tested, the Genie Nano and Basler Ace have the best performance; however, they are also the two largest of the machine vision cameras tested. The smaller cameras (Basler Dart and Ximea XiMU) have shown that despite their diminutive size, they are capable of taking PSP measurements, especially where large pressure changes will be present. These smaller cameras can help take measurements using PSP where larger cameras cannot be used and could also be used on real vehicles for on-board measurements.

## Figures and Tables

**Table 1:** Camera comparison.

Camera Name	Size (mm)	Resolution (Pixels)	ADC (Bits)	Quantum Efficiency	Shutter Type	Frame Rate (Hz)
PCO.1600(mod)	200 × 70 × 84	1600 × 1200	14	60% @ 550 nm	Global	4.8
Basler ACE acA2040-90um	29 × 29 × 47	2048 × 2048	8, 12	60% @550 nm	Global or Progressive	90
Basler DART daA1600-60um	20 × 29 × 20	1600 × 1200	8, 12	48% @ 510 nm	Global or Progressive	60
Teledyne DALSA Genie Nano M1920	44 × 29 × 41	1920 × 1200	8, 12	65% @ 510 nm	Global	38
Ximea Sub-Miniature XiMU9PM	15 × 15 × 9	2594 × 1944	8, 12	60% @ 500 nm	Progressive	5.8

**Table 2:** Exposure times for different tests.

Camera	Linearity Max Exposure Time (ms)	Linearity Min Exposure Time (ms)	PSP Calibration Exposure Time (ms)
PCO.1600	3.8	0.2	1.5
Basler Ace	3.0	0.3	2.0
Basler Dart	3.0	0.3	2.8
Genie Nano	3.0	0.3	6.0
Ximea XiMU	3.0	0.3	7.0

**Table 3:** Linearity results for different cameras.

Camera	Gradient	Offset	R^2^	Non-Linearity (%)
PCO.1600	3941.9	460.8	1.0000	0.36
Basler Ace	1272.0	−8.8	0.9997	1.93
Basler Dart	1244.4	−8.2	0.9999	0.81
Genie Nano	1240.5	42.6	0.9998	1.51
Ximea XiMU	1239.1	−269.9	1.0000	0.23

**Table 4:** Comparison of PSP Calibration Parameters.

Camera	A	B	Temp (K)
PCO.1600	0.176	0.683	273
	0.192	0.798	293
	0.218	0.943	313
Basler Ace	0.149	0.715	273
	0.161	0.835	293
	0.176	0.976	313
Basler Dart	0.171	0.715	273
	0.182	0.810	293
	0.200	0.963	313
Genie Nano	0.186	0.722	273
	0.190	0.799	293
	0.214	0.967	313
Ximea XiMU	0.184	0.699	273
	0.198	0.791	293
	0.256	0.929	313

**Table 5:** Uncertainty estimation per camera.

Camera	A	B
PCO.1600	±0.010	±0.015
Basler Ace	±0.024	±0.016
Basler Dart	±0.033	±0.017
Genie Nano	±0.011	±0.014
Ximea XiMU	±0.028	±0.016
